# What about the teachers? Developing and validating a physical literacy assessment scale for primary and secondary physical educators

**DOI:** 10.3389/fpsyg.2026.1989587

**Published:** 2026-10-29

**Authors:** Yusen Xu, Zhenyu Ren

**Affiliations:** School of Physical Education, Yanshan University, Qinhuangdao, Hebei, China

**Keywords:** assessment tools, physical education, physical educators, physical literacy, scale development

## Abstract

**Purpose:**

Physical education teachers bear the primary responsibility for fostering students’ physical literacy; however, there is currently a lack of assessment tools specifically designed to measure the physical literacy of primary and secondary physical educators (PET). This study aims to develop and validate a physical literacy assessment scale for primary and secondary physical educators (PLAS-PET).

**Methods:**

Domains and items were generated from a systematic literature review and a nominal group technique with three experts. A two-round Delphi panel of 14 experts assessed content validity. A total of 832 physical educators completed the survey; 780 valid questionnaires (93.7%) were randomly divided into two subsamples (*n* = 390 each). Subsample 1 was used for item analysis and exploratory factor analysis (EFA); subsample 2 was used for confirmatory factor analysis (CFA), reliability, and validity testing. Reliability included Cronbach’s *α*, Spearman–Brown split-half coefficients, and McDonald’s *ω*. Validity included content validity index (I-CVI, S-CVI), average variance extracted (AVE), composite reliability (CR), and the Fornell–Larcker criterion.

**Results:**

The final PLAS-PET contained 20 items in four domains: physical affectivity, physical skill, physical cognition, and physical conduct. Item analysis showed all 30 items were significant (*p* < 0.001; critical ratios = 8.523–29.443). EFA extracted four factors explaining 75.717% of the variance. CFA supported a first-order four-factor model: RMSEA = 0.071, *χ*^2^/df = 3.116, GFI = 0.874, CFI = 0.935, IFI = 0.935, TLI = 0.925, SRMR = 0.072. Cronbach’s *α* was 0.958, and split-half reliability was 0.911. Subscale *α*, split-half, and *ω* ranged from 0.850–0.940, 0.770–0.921, and 0.753–0.917, respectively. I-CVI ranged from 0.800 to 1.000, S-CVI/UA = 0.800, and S-CVI/Ave = 0.960. AVE (0.651–0.682) and CR (0.859–0.938) supported convergent validity; √AVE (0.807–0.826) exceeded inter-factor correlations.

**Conclusion:**

The PLAS-PET demonstrates high reliability and validity, making it an effective tool for measuring the physical literacy of PET. Future validation studies in diverse countries and regions are necessary to enhance the applicability of assessment tools.

## 1 Introduction

Physical literacy (PL) lies at the heart of physical education instruction, embodying both the goals of school physical education and the educational value of the discipline (Wickens and Parker, 2024). It is widely recognized globally and has become a central objective and integral component of physical education in many international organizations and developed countries (Durden-Myers and Whitehead, 2018). For instance, the “International Position Statement on Physical Education” published by the International Council of Sport Science and Physical Education (ICSSPE) in 2010 (International Council of Sport Science and Physical Education, 2010), the “International Charter on Physical Education, Physical Activity, and Sport” revised by UNESCO in 2015 (UNESCO, 2015), and the latest edition of the “K–12 National Physical Education Standards” released by SHAPE America in 2024 all identify PL as a primary goal of physical education (Seymour et al., 2024). Additionally, China’s Outline for Building a Sports Powerhouse, issued in 2019, highlights promoting PL among youth as a key component of school physical education (General Office of the State Council, 2019). It is therefore evident that the theory of PL has already had a significant impact on the practical implementation of school physical education worldwide.

The current academic discussion on PL centers on a comprehensive concept reinterpreted by British scholar Margaret Whitehead in 1993 (Whitehead, 2001). This concept is grounded in monism, phenomenology within monism, and existentialism as part of the phenomenological movement (Shearer et al., 2018). It focuses on the development of individuals at multiple levels, including physical, psychological, and social, and advocates that everyone should participate in physical activity throughout their lives (Whitehead, 2010). Whitehead’s articulation of PL has profoundly influenced physical education policies and practices worldwide, serving as a key reference and model for reforms in many countries (Stoddart et al., 2023; Till et al., 2025). Existing research demonstrates that PL is a crucial foundation for the well-rounded development of adolescents and a significant factor in achieving substantial health benefits (Zhang and Yun, 2025). Fostering PL promotes the formation of lifelong physical activity habits and serves as a vital starting point for enhancing students’ physical and mental health, as well as their overall quality of life (Liu et al., 2026). It is evident that how school physical education programs cultivate and develop students’ PL has become a major global concern. Regarding the cultivation of students’ PL, existing research has explored its development from perspectives such as the design of physical education classroom instruction (Xie et al., 2025), the practical application of teaching models (Choi et al., 2024), and the development of assessment tools for PL among elementary, secondary, and college students (Liu et al., 2026; Mota et al., 2023). These studies reveal the demands that PL development places on physical education instruction. Although these research findings have promoted curriculum reform based on PL, there has been limited discussion of PL development from the perspective of physical education teachers. Specifically, how to assess physical education teachers’ own levels of PL to better promote students’ PL. This highlights the need for further research on PL at the teacher level.

As the primary practitioners responsible for fostering students’ PL (Choi et al., 2021), the PL levels of primary and secondary physical educators directly determine whether physical education courses can effectively cultivate students’ PL and to what extent the instructional objectives of these courses can be achieved (Stoddart and Selanders, 2022; Silverman and Mercier, 2015). In other words, as key agents in promoting students’ PL, the PL levels of primary and secondary school physical education teachers directly influence their enthusiasm, initiative, and creativity in engaging with physical education practices (Carreiro-da-Costa, 2019). This makes their PL a crucial driving force for the in-depth development and effective implementation of physical education. Therefore, within the context of literacy-based teaching, cultivating the PL of primary and secondary school physical education teachers is an urgent priority. A methodical and scientific evaluation tool serves as a tangible indicator of the current status of physical education teachers’ PL development in primary and secondary schools (Sepahvand et al., 2025). It also provides an effective way to identify existing PL gaps and propose strategies for improvement (Yin et al., 2024). However, a review of global research on PL reveals that studies on physical education teachers’ PL are still in their infancy (Hernaiz-Sánchez et al., 2025).

A critical examination of existing PL assessment tools reveals three interrelated limitations that collectively justify the need for a dedicated instrument for physical educators. (1) The majority of validated PL instruments have been developed for children and adolescents (Blanchard et al., 2020; Matsunaga et al., 2025), such as the Canadian Assessment of Physical Literacy (CAPL) and the Passport for Life, which are contextually and developmentally inappropriate for adult professionals. (2) The Perceived Physical Literacy Instrument (PPLI), developed by Sum et al. for PE teachers, contains only nine items, rather than reflecting the full multidimensional nature of PL (Sum et al., 2016). A recent systematic review concluded that no comprehensive tool currently exists to assess PE teachers’ PL across all relevant domains, and that existing instruments have been adapted from student or general adult populations rather than developed specifically for the teaching profession (Boldovskaia et al., 2023; Yin et al., 2024). (3) While Whitehead’s framework identifies four domains, affective, physical, cognitive, and behavioral many existing instruments emphasize the affective and cognitive domains, giving less attention to the physical domain and especially the behavioral domain (Edwards et al., 2017), which is particularly consequential for teachers who serve as role models of physical activity. These limitations point to a clear and pressing need for a psychometrically sound, domain-comprehensive PL assessment tool tailored specifically to primary and secondary physical educators.

To address the identified research gap, there is an urgent need to develop a PL assessment tool specifically tailored for primary and secondary physical educators. It is worth noting that, currently, no assessment tool can comprehensively and differentially measure all key attributes (physical, affective, behavioral, etc.) according to Whitehead’s definition, nor quantitatively score the PL of primary and secondary physical educators. Therefore, the objective of this study is to develop the physical literacy assessment scale for primary and secondary physical educators (PLAS-PET). To a certain extent, the tool is user-friendly for self-assessment and can save both time and costs in large-scale evaluations. This study contributes to the quantitative evaluation of PL among primary and secondary physical educators, offering valuable insights to advance global reforms in school physical education curricula effectively.

## 2 Methods

### 2.1 Flowchart of the scale development and validation process

The development and validation of the PLAS-PET followed a systematic two-phase procedure based on educational measurement theory (Figure 1). The first phase, scale development, involved item generation, item modification, and item reduction. Initial items were generated through a literature review and nominal group technique with three experts. The item pool was then refined through a two-round Delphi consultation with 14 experts, and content validity was assessed using item-level content validity index (I-CVI), scale-level content validity index (S-CVI), and S-CVI/UA. After participant sampling, item analysis was conducted to identify and remove items with inadequate discrimination or homogeneity. The second phase, scale validation, focused on reliability and validity. Construct validity was examined through exploratory factor analysis (EFA) and confirmatory factor analysis (CFA). Reliability was evaluated using Cronbach’s alpha, Spearman–Brown split-half coefficient, and the McDonald’s *ω*. Convergent and structural validity were further assessed using average variance extracted (AVE), composite reliability (CR), and CFA model fit indices. Discriminant validity was tested using the Fornell–Larcker criterion. This stepwise procedure ensured that the PLAS-PET was developed through both expert-based qualitative refinement and sample-based quantitative psychometric testing.

**Figure 1:**
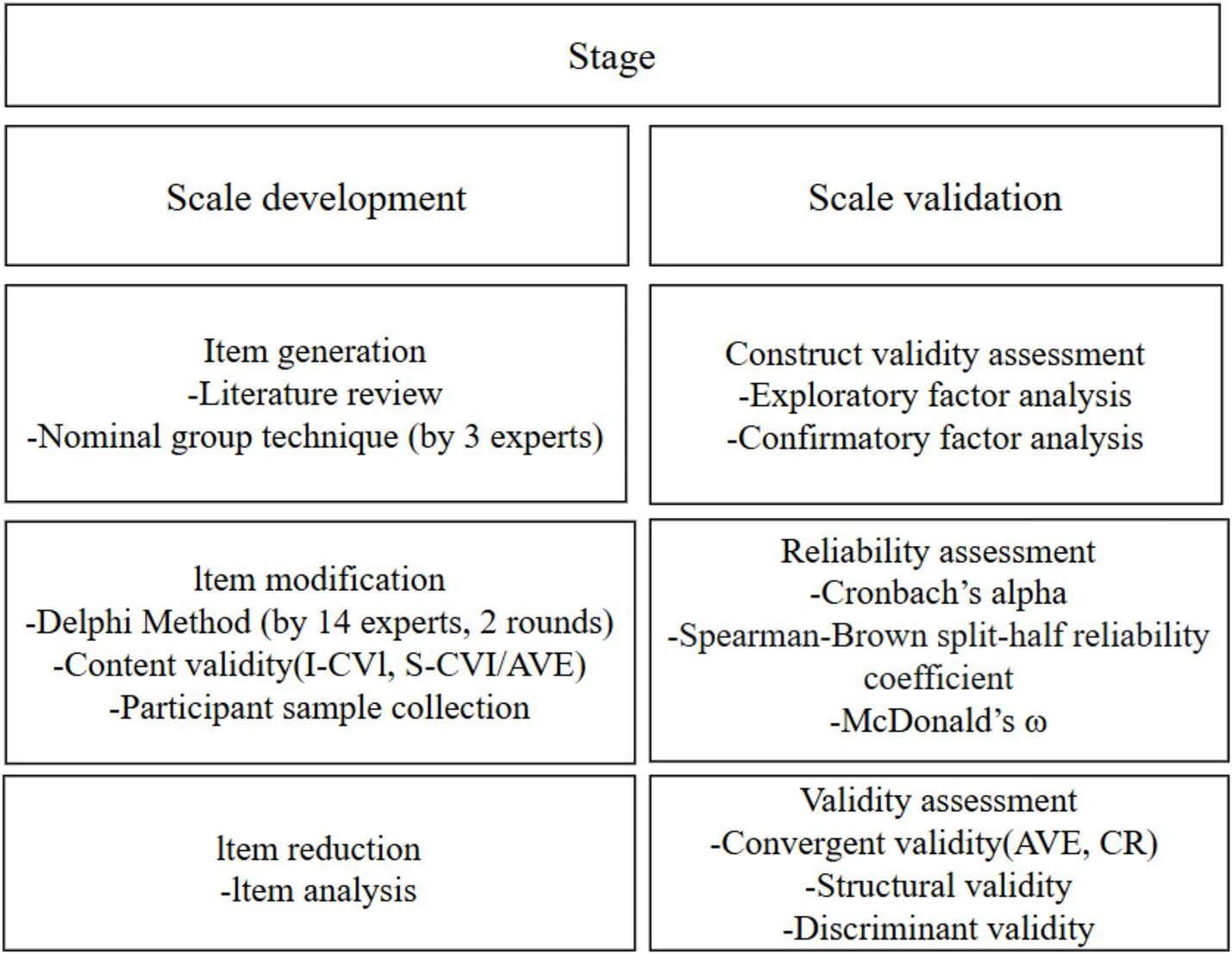
Scale development and validation flowchart.

### 2.2 Participants

All participants in this study were frontline physical education teachers in primary and secondary schools. Between June and August 2026, the researchers employed convenience sampling and used “QuestionnaireStar” software to distribute the initial PLAS-PET (survey questionnaire) online to physical education teachers across five Chinese provinces and municipalities: Beijing, Hebei, Liaoning, Hubei, and Shanghai. Participation was voluntary and without incentives. Teachers were eligible if they (1) were currently teaching PE with classroom responsibilities, (2) could read Chinese, and (3) provided informed consent. No additional school-level inclusion criteria were applied except that schools had to be primary, junior high, or senior high schools. A total of 832 participants completed the initial PLAS-PET. The questionnaire response rate was 100%. Questionnaires were excluded for (1) missing responses (defined as more than 20% of scale items missing); (2) some invalid questionnaires were excluded due to missing responses or logical inconsistencies (All the options entered were consistent). Ultimately, 780 valid questionnaires were retained; the effective rate of the questionnaire is 93.7% (780/832). Because the questionnaire data collected in this study are not used for mean comparisons in the current-situation analysis, the sample of teachers may be drawn from a single institution. The final sample (*n* = 780) was randomly divided into two subsamples using the random case selection function in SPSS software. Subsample 1 (*n* = 390) was primarily used for item analysis and EFA of the PLAS-PET. This group included 217 males (55.6%) and 173 females (44.4%); 128 elementary school teachers (32.8%), 147 middle school teachers (37.7%), and 115 high school teachers (29.5%), with an average teaching experience of 7.2 years. Subsample 2 (*n* = 390) was primarily used for CFA and reliability and validity testing of the PLAS-PET. This group comprised 207 males (53.1%) and 183 females (46.9%); 134 elementary school teachers (34.4%), 133 junior high school teachers (34.1%), and 123 high school teachers (31.5%), with an average teaching experience of 6.1 years. This study was approved by the Institutional Ethics Review Committee of the School of Physical Education at Yanshan University, and an official approval document bearing the university seal was obtained. The researchers provided a detailed explanation of the study’s objectives, procedures, potential risks, and benefits, and the participants were given sufficient time to ask questions. Written informed consent was obtained from all participants before data collection. Participants’ right to confidentiality of their personal information and their right to withdraw from the questionnaire at any time were protected. All procedures were conducted in accordance with the Declaration of Helsinki.

### 2.3 Literature review

The literature review aimed to clarify the fundamental content structure of PL, clearly identifying its concepts, characteristics, appropriate populations, and various assessment tools. A systematic search was conducted in databases such as Web of Science, Scopus, PubMed, and CNKI. The search strategy combined keywords such as “PL” and “physical educators,” whilst also incorporating free-text terms and controlled vocabularies to enhance the sensitivity of the search. Only English- and Chinese-language publications from 2006 to 2026 were included to ensure their contemporary relevance. To minimize bias, all relevant publications were taken into account, regardless of research methodology (qualitative, survey, systematic review, or quantitative), article type (theoretical or empirical), or source. Following a comprehensive analysis of the literature and discussions with three experts from the technical group, a consensus was reached on the definition of PL.

### 2.4 The Delphi method

Based on an analysis of previous research, the Delphi method is a widely used approach for developing scale frameworks (Sterkenburg et al., 2025). Its primary purpose is to eliminate mutual influence among participating experts by repeatedly soliciting their opinions anonymously and synthesizing the results to derive predictive outcomes that reflect the experts’ views. In this study, through two rounds of expert consultation, we gathered and compiled evaluations from 14 senior experts in the fields of school physical education and physical education teacher development regarding the preliminary evaluation indicators and items of the PLAS-PET. Expert selection criteria. Experts were eligible if they met at least two of the following criteria: (1) holding a doctoral degree in physical education, sport pedagogy, or educational measurement; (2) having published at least five peer-reviewed articles on PL or teacher education in the past 5 years; (3) having at least 10 years of experience in PE teacher training or curriculum development; and (4) having prior experience with scale development or Delphi methodology. Fourteen experts who met these criteria were invited, all of whom agreed to participate. The panel included 7 professors, 4 associate professors, and 3 lecturers, all holding Ph.D. degrees. The 14-member Delphi panel then reviewed the item pool in two rounds, providing content validity evidence and recommending modifications or deletions. Fourteen experts participated in two rounds. The two rounds served distinct purposes.

Round 1 was primarily qualitative. In this study, the initial scale was adapted into an expert consultation questionnaire, and the first round of expert consultation was conducted. Using the Questionnaire Star software, 14 experts were invited to perform a qualitative assessment of each evaluation indicator. The assessment criteria primarily included whether the indicators were meaningful, clearly expressed, and easy to understand; whether they were obscure or difficult to interpret; whether any ambiguities existed; whether they applied to the study population; and whether the various dimensions were relatively independent of one another. Predefined rules for item modification and deletion. Prior to data analysis, the research team established the following decision rules: (1) items with ≥30% of experts recommending deletion were to be removed; and (2) items receiving qualitative suggestions for rewording without substantive disagreement were to be revised and retained for the second round (Groves et al., 2011). Disagreements were resolved through discussion with the research team, and items receiving qualitative suggestions for rewording without substantive disagreement were revised and retained for the second round.

Round 2 was quantitative. The Delphi expert consultation method primarily assesses content validity by examining the expert engagement coefficient and the expert authority coefficient (Cr). The expert engagement coefficient reflects the degree of experts’ interest and cooperation in the study, typically measured by the questionnaire response rate. Scholars agree that an expert response rate of 70% or higher indicates a high level of engagement, and such results are considered acceptable (Denecke et al., 2024). The Cr significantly influences the quality of the consultation results. Cr is determined by the coefficient of experts’ judgment basis (Ca) for the indicators and the coefficient of experts’ familiarity (Cs) with the issues, each assigned a value across five levels. The calculation formula is Cr = (Ca + Cs)/2. Academic research indicates that when Cr exceeds 0.7, it reflects a high level of advisory authority within the expert group; moreover, the higher the Cr value, the greater the experts’ authority (Chen et al., 2022). The expert coefficient of variation (CV) represents the degree of coordination amongst experts regarding a particular indicator; the lower the CV, the higher the degree of coordination. It is calculated as follows: CV = standard deviation/mean × 100 per cent. A CV of ≤0.25 is often taken as the benchmark for a good degree of coordination amongst experts. A high degree of coordination is an important condition for reaching consensus, but it is not a sufficient condition. The degree of consensus is the result of a comprehensive assessment of the degree of central tendency (mean), the degree of coordination, and other factors (such as the authority coefficient) (Nasa et al., 2021).

### 2.5 Exploratory factor analysis

The purpose of EFA is to reduce a large number of complex variables into a smaller set of common factors (latent dimensions), thereby revealing the underlying structure of the original variables. A larger sample size in EFA generally leads to more stable and reliable results. Many researchers recommend a sample size to item ratio of at least 5:1, ideally 10:1 or higher, with a minimum total sample size of 100 participants (Guadagnoli and Velicer, 1988; Nunnally, 1978). Before conducting factor extraction, two statistical tests must be performed to determine whether the data are suitable for EFA: (1) The Kaiser-Meyer-Olkin (KMO) test, which measures the adequacy of correlations among variables and ranges from 0 to 1. A KMO value below 0.5 indicates that the data are unsuitable for factor analysis; values above 0.8 indicate suitability, and values above 0.9 indicate excellent suitability (Han et al., 2023). (2) Bartlett’s Test of Sphericity, which assesses whether the correlation matrix is an identity matrix (i.e., whether the variables are independent). A significant *p*-value (*p* < 0.05) indicates sufficient correlations among variables to justify factor extraction (Williams et al., 2010).

In the social sciences, if the cumulative variance explained by the extracted common factors reaches 60% or higher, the factors are considered reliable; if it reaches 50% or higher, the factor analysis results are deemed acceptable (Williams et al., 2010). An EFA was conducted to identify potential common factors. Using principal component extraction, the number of common factors was determined based on the eigenvalue (>1) and the scree plot (Kaiser, 1960). Since the correlation between the two factors was not close to zero, promax rotation was used as suggested by Meyers et al. (2016). Get the factor loadings of the rotated items. Items were deleted if the rotated factor loadings were below 0.50 or if there were cross-loadings (i.e., the items loaded close together on both factors). After completing these steps, the EFA was finalized, resulting in the PLAS-PET.

### 2.6 Confirmatory factor analysis

CFA is a statistical technique based on structural equation modeling used to test whether the relationships between observed variables and latent factors align with a predefined theoretical model. The total sample size for CFA should be at least five times the number of items, preferably 10 times or more, and must include at least 200 participants (Clark and Watson, 1995) Once these requirements are met, the maximum likelihood (ML) estimation method in AMOS 24.0 is employed to analyze the fit of the initial structural equation model after iterative convergence. CFA was conducted in AMOS 24.0 using ML estimation. The five-point Likert items were treated as approximately continuous observed indicators rather than as ordered categorical variables. This is a common and defensible approach when items have at least five response categories and distributions are not severely non-normal (Rhemtulla et al., 2012). ML provides consistent parameter estimates and is relatively robust to moderate violations of normality, particularly in large samples (Bentler and Chou, 1987). The total sample size (*N* = 780) and the subsample size (*n* = 390) exceed common requirements for ML-based CFA.

In CFA, the primary measures of model fit include the Chi-Square Divided by Degrees of Freedom (*χ*^2^/df), Root Mean Square Error of Approximation (RMSEA), Goodness-of-Fit Index (GFI), Incremental Fit Index (IFI), Tucker-Lewis Index (TLI), Comparative Fit Index (CFI), and Standardized Root Mean Square Residual (SRMR) (Boateng et al., 2018). Generally, a smaller *χ*^2^/df ratio indicates a better and more parsimonious model fit (Raykov and Marcoulides, 2011). A ratio less than 3 signifies an ideal fit, while a ratio below 5 is still considered acceptable. An RMSEA value below 0.05 indicates excellent fit; values between 0.05 and 0.08 indicate good fit; values between 0.08 and 0.10 are acceptable; and values above 0.10 suggest poor fit (Browne and Cudeck, 1993). The GFI, IFI, TLI, and CFI should all exceed 0.90 to indicate acceptable model fit; however, individual indices slightly above 0.80 and just below 0.90 are also considered acceptable (Hu and Bentler, 1999). A SRMR of <0.08 indicates that the model fits the data acceptably (Boateng et al., 2018).

### 2.7 Reliability analysis

In this study, the reliability of the PLAS-PET was primarily evaluated using Cronbach’s alpha coefficient, a measure of internal consistency, and the Spearman–Brown split-half reliability coefficient. Cronbach’s alpha coefficient accurately reflects the degree of consistency among test items and the quality of the scale’s internal structure. The evaluation criteria are as follows: a value less than 0.5 indicates low reliability; a value between 0.7 and 0.8 is considered acceptable; a value between 0.8 and 0.9 indicates an ideal scale with high reliability; and a value greater than 0.9 indicates an excellent scale with outstanding reliability (Cronbach, 1951). The Spearman–Brown split-half reliability coefficient is an important indicator used to assess the internal consistency of a measurement instrument. Its values range from −1 to 1; the higher the value, the better the internal consistency of the scale. In practical research, a value of ≥0.70 is the most widely accepted standard. If the coefficient is below 0.60, the scale’s reliability is generally considered poor (Charter, 2000). In addition, McDonald’s *ω* (McDonald, 2013) was computed from the standardized factor loadings and residual variances obtained in the CFA. Unlike Cronbach’s *α*, *ω* does not assume equal factor loadings across items and provides a model-based reliability estimate that is more appropriate when item loadings are heterogeneous (Rodriguez et al., 2016).

### 2.8 Validity analysis

In this study, the primary validity measures were content validity, convergent validity, and discriminant validity. First, regarding content validity, this primarily involved the I-CVI and the S-CVI. Fourteen senior experts were invited to assess the relevance of the items to their corresponding evaluation indicators (second round of Delphi expert consultation). The I-CVI for each item is calculated as the number of experts who awarded a score of 4 or 5 ÷ the total number of experts involved in the evaluation. S-CVI/UA is calculated as the number of items for which all experts awarded a score of 4 or 5 ÷ the total number of items; S-CVI/Ave is the arithmetic mean of the I-CVI values for all items. It is generally accepted in the academic community that an I-CVI of at least 0.78 and an S-CVI of at least 0.80 indicate good content validity for a scale (Polit et al., 2007). Second, regarding convergent validity, the AVE and CR were used to verify the scale’s convergent validity. Hair et al. noted that a scale demonstrates good convergent validity only when it simultaneously meets the following criteria: (1) the AVE is greater than 0.500, and (2) the CR is greater than 0.700 (Hair et al., 2019). Discriminant validity was tested using the Fornell–Larcker criterion. The square roots of the AVEs for each latent variable were placed along the diagonal of the correlation matrix, whilst the off-diagonal elements represented the correlation coefficients between the latent variables. If the square root of the AVE for each latent variable is greater than the absolute value of its correlation coefficient with any other latent variable, this indicates that the variance explained by that latent variable within its own set of indicators is greater than the variance shared with other latent variables, thereby supporting discriminant validity. Furthermore, as the AVE for each latent variable is greater than 0.50, this indicates that the scale possesses acceptable convergent validity. Model fit indices derived from CFA can be used to further confirm the scale’s construct validity.

## 3 Results

### 3.1 Definition and boundary of physical literacy

The research team reviewed existing literature on PL and compared scholars’ interpretations of the concept worldwide. Although numerous international definitions of PL exist (Carcamo-Oyarzun et al., 2025; Hyndman and Pill, 2018), this study focuses exclusively on the concept proposed by Whitehead, as it serves as the origin and foundation of current research in this field (Edwards et al., 2017; Edwards et al., 2018). Whitehead defines PL as the motivation, confidence, physical competence, knowledge, and understanding that enable individuals to value and responsibly engage in physical activity throughout their lives (Whitehead, 2001). According to her framework, PL comprises four domains: the affective domain (motivation and confidence), the skill domain (physical competence), the cognitive domain (knowledge and understanding), and the behavioral domain (responsible participation in physical activity). Although Whitehead’s conceptualization of PL was originally proposed for individuals across the lifespan, its application to primary and secondary physical educators requires professional contextualization (Whitehead, 2010). In this study, physical educators’ PL is defined as the motivation, confidence, physical competence, knowledge and understanding, and responsible physical activity behaviors that enable physical educators to value, participate in, and model lifelong physical activity and to enact these attributes in school physical education. It is not defined as generic teaching efficacy, pedagogical content knowledge, or students’ PL. At the same time, the research team conducted a survey and analysis of the actual teaching practices of frontline physical education teachers. There was consensus that physical education teachers’ words and actions subtly influence students; teachers’ attitudes (behavior and moral character) and their restraint from arbitrarily reprimanding students can effectively alleviate students’ fear and social anxiety during physical activities (Huéscar Hernández et al., 2020), thereby improving learning efficiency and fostering PL. The four domains are therefore operationalized as follows: physical affectivity, physical skill, physical cognition, and physical conduct. Physical affectivity refers to teachers’ own motivation, confidence, and enjoyment in physical activity, with emotional engagement in teaching acceptable only as role-modeling of that personal affect. Physical skill refers to teachers’ own movement competence, practice, and demonstration of skills. Physical cognition refers to teachers’ knowledge and understanding of physical activity, health, and their own PL development, not pedagogical content knowledge or student learning. Physical conduct refers to teachers’ own responsible physical activity behavior and their role-modeling of that behavior. This definition distinguishes physical educators’ PL from related but distinct constructs and provides a clear boundary for item generation and validation (Table 1).

**Table 1:** Concept mapping table of primary and secondary physical educators’ physical literacy.

Concept	Whitehead original dimension	The connotation of teachers’ physical literacy
PA	Affective	Motivation, confidence, and emotional engagement in physical activity and teaching.
PS	Skill	Movement competence, demonstration, and active participation.
PC	Cognitive	Knowledge and understanding of PL, curriculum, and pedagogy.
PCD	Behavioral	Responsible, ethical, and lifelong physical activity behaviors that model PL for students.

PA, Physical affectivity; PS, Physical skill; PC, Physical cognition; PCD, Physical conduct.

In summary, drawing on the elements of PL and considering the actual teaching practices of frontline physical education teachers in primary and secondary schools, the researchers identified physical affectivity, physical skill, physical cognition, and physical conduct as the core elements of PL for these teachers. These elements constitute the four domains of the PLAS-PET. A nominal group technique involving three experts (all specializing in school sports measurement) was used to refine the definitions of the domains and review their clarity; ultimately, the researchers and the three experts reached a consensus on the four domains of PL.

### 3.2 Design of physical literacy assessment scale for primary and secondary physical educators

First, the four core elements constituting the PL of primary and secondary physical educators were identified as first-level assessment indicators (domains). Second, based on the connotations of each first-level assessment indicator, corresponding observable variables (i.e., test items) were selected. These variables were primarily obtained through two approaches: first, by adapting items from teacher literacy assessment tools in other academic disciplines, with modifications to contextual settings and core terminology; and second, by referencing relevant content from global PL assessment tools. Items were retained if they demonstrated conceptual relevance to physical educators’ professional context and were deemed suitable for adaptation.

It is particularly important to emphasize that the PL of primary and secondary physical educators is demonstrated in the teaching context; teachers’ physical activity behaviors are, by their very nature, exemplary and context-embedded (Yıldızer and Munusturlar, 2022), which differs fundamentally from general teaching efficacy or teaching knowledge. For physical education teachers as a group, several PLAS-PET items (particularly in the physical conduct domain) reference teaching-related behaviors such as “I will incorporate content related to ethics and spirit into my physical education lessons.” This reflects the theoretical position that physical educators enact responsible physical activity behaviors not only in personal life but also through professional role modeling (Bandura, 2001). Whitehead’s conceptualization emphasizes that PL individuals “assist others in acquiring these skills” (Whitehead, 2010), which for teachers necessarily involves the pedagogical context. When designing items for an assessment scale, failure to consider the teaching context of physical education teachers may result in the physical education literacy of this specific group being indistinguishable from that of other groups (such as adolescents or adults) (Essiet et al., 2022). From this perspective, it can be understood that, to some extent, this will lead to consistency between the PL assessment scales used for physical education teachers and those designed for adolescents and adults. Considering this not only clarifies the scope of PL for primary and secondary physical educators, but also highlights its distinctive characteristics. Thus, the teaching-referenced items in the PLAS-PET capture the enactment of PL in the professional role, not generic pedagogical competence per se. The PLAS-PET, preliminarily developed in this study, comprises four domains: physical affectivity, physical skill, physical cognition, and physical conduct, with each domain including 8 items, making a total of 32 items. Further details regarding the attributes and rationale for the items selected in PLAS-PET to measure the physical education literacy of primary and secondary school PE teachers are provided in Supplementary Table S1 (Appendix 1).

### 3.3 Delphi expert consultation

Results of the first round of consultation. A total of 14 questionnaires were distributed during this round, with 14 returned and all 14 deemed valid, resulting in a 100% response rate and indicating a high level of expert engagement. Upon reviewing the returned questionnaires, it was found that all four top-level evaluation indicators received approval from all 14 experts. However, some experts offered the following suggestions regarding the measurement items: several recommended adding an item on “the application of teaching methods” under the physical cognition domain, while others suggested including items on “caring for students” and “taking responsibility for students” under the physical conduct domain. Additionally, some experts noted that in the physical education skills domain, the statements “I demonstrate technical movements in every class” and “I demonstrate technical movements to students when correcting incorrect techniques” were redundant and violated the principle of independence. Furthermore, in the physical affectivity domain, the frequency of the statement “I frequently inquire about students’ academic and personal lives” was unclear and lacked scientific rigor. In response to these comments, after reviewing relevant materials and engaging in repeated discussions with various experts, this study decided to adopt these suggestions and remove the problematic items. Consequently, the PLAS-PET now comprises four first-level evaluation indicators and 30 measurement items.

Results of the second round of consultation. The PL assessment indicators for primary and secondary physical educators, which had undergone the first round of revisions and adjustments, were compiled into a 5-point Likert scale. We asked each expert to assign a score to each evaluation indicator on a scale of 5 (very important), 4 (fairly important), 3 (moderately important), 2 (fairly unimportant), and 1 (very unimportant). Fourteen questionnaires were distributed in this round, with 14 returned and 14 deemed valid, resulting in a 100% response rate. The Ca ranged from 0.8 to 1.0, indicating that theoretical foundations and practical experience largely influenced the experts’ opinions. The Cs ranged from 0.8 to 1.0, indicating that the experts had a high level of familiarity with the field. The Cr ranged from 0.8 to 1.0, with an average value of 0.899, which also reflects the experts’ high level of authority in this field. The results of this round were as follows: no new dimensions or themes emerged; no items met the deletion threshold of ≥30 percent; and the experts did not propose any further substantive amendments. The experts’ opinions remained stable between the two rounds. The mean importance scores for the 30 initial items ranged from 4.29 to 4.79 (all ≥4.0), with CV ranging from 0.09 to 0.17 (all ≤0.25) (Appendix 2). Comprehensive consideration, all items met the pre-defined consensus retention criteria (Mean ≥4.0 and CV ≤ 0.25); consequently, all 30 items were retained following the second round of the Delphi study. Limiting the number of rounds helps maintain the quality of expert participation and responses, as a prolonged process may lead to diminishing returns and increased participant fatigue (O’Malley et al., 2023). The two-round design aligns with methodological recommendations that Delphi consensus requires at least two rounds; many studies have achieved consensus after two rounds (Nasa et al., 2021).

### 3.4 Items analysis

First, a critical ratio analysis was conducted on the 30 initial items of the PLAS-PET. The scores for the 30 items were summed and ranked from highest to lowest. We classified the top 27% of participants as the high-score group and the bottom 27% as the low-score group. We then performed independent-samples *t*-tests for each item across the two groups. The critical ratio values ranged from 8.523 to 29.443, and all items reached a highly significant level (*p* < 0.001). Therefore, all 30 items were retained at this stage. This indicates that the items had good discriminative ability between high- and low-scoring respondents.

Next, a homogeneity test was conducted using the corrected item-total correlation. Each item was correlated with the sum of the remaining items rather than with the total score that included the item itself. This procedure prevents artificial inflation of the item-total correlation due to the item’s own contribution to the total score (Henrysson, 1963). A correlation coefficient below 0.40 would indicate that the item has only a weak relationship with the scale’s construct and lacks homogeneity with the overall scale; such an item should be deleted (Boateng et al., 2018). The results showed that the corrected item-total correlations for all 30 items were greater than 0.40. The individual values are reported in Supplementary Table S4 (Appendix 4). In summary, all 30 items passed the item analysis and were retained for EFA.

### 3.5 Results of exploratory factor analysis

The item-to-respondent ratio for Subsample 1 (*n* = 390) and the PLAS-PET was greater than 10:1, thus meeting the sample size requirements for educational measurement and satisfying the prerequisites for EFA. First, the data suitability was assessed using the KMO and Bartlett’s sphericity tests. The results showed a KMO value of 0.960 and an approximate chi-square value of 6,670.767 for Bartlett’s sphericity test (*p* < 0.001), indicating that the sample was highly suitable for factor analysis (Table 2).

**Table 2:** KMO measures and Bartlett’s test of sphericity.

KMO Quantity of Sample Suitability		0.960
	Approximate chi-square value	6670.767
Bartlett’s test of sphericity	Degrees of freedom	435
	Significance	0.000

The results showed that after extracting four common factors, the cumulative variance contribution rate stabilized, and the increase in the variance contribution rate from subsequent factor extractions decreased significantly, indicating that the four-factor model effectively explained the structural characteristics of the scale. Simultaneously, 10 items were removed (factor loadings below 0.5) (Appendices 1, 3), ultimately resulting in a PLAS-PET consisting of 20 items and 4 factors (Table 3). The four extracted factors collectively explained 75.717% of the total variance.

**Table 3:** Results of exploratory factor analysis.

Domains	Items	F1	F2	F3	F4
PA	Q1	0.656			
	Q2	0.739			
	Q3	0.792			
PS	Q4		0.718		
	Q5		0.760		
	Q6		0.726		
	Q7		0.674		
	Q8		0.651		
	Q9		0.774		
PC	Q10			0.746	
	Q11			0.704	
	Q12			0.843	
	Q13			0.858	
PCD	Q14				0.592
	Q15				0.764
	Q16				0.718
	Q17				0.722
	Q18				0.654
	Q19				0.644
	Q20				0.610

PA, Physical affectivity; PS, Physical skill; PC, Physical cognition; PCD, Physical conduct.

Based on the results of EFA, the four factors were named as follows: Factor 1, comprising items Q1, Q2, and Q3, was named “physical affectivity”; Factor 2, comprising items Q4, Q5, Q6, Q7, Q8, and Q9, was named “physical skill”; Factor 3, comprising items Q10, Q11, Q12, Q13, and was named “physical cognition”; Factor 4 comprises items Q14, Q15, Q16, Q17, Q18, Q19, and Q20, and was named “physical conduct.” Ultimately, these four factors and 20 items together form the PLAS-PET (Table 4). The PLAS-PET consists of 20 items scored from 1 to 5. Because the four domains contain unequal numbers of items (PA = 3, PS = 6, PC = 4, PCD = 7), a simple sum of all 20 items would implicitly give greater weight to domains with more items. We therefore recommend that domain scores be computed as the mean of the items within each domain, and that a total score be computed as the average of the four domain means. This ensures that each domain contributes equally to the composite, consistent with the theoretical position that all four domains are constitutive of physical educators’ PL (Whitehead, 2010). This equal-domain-weighting approach avoids the implicit over-representation of the domain. Equal weighting is justified when each domain is conceptually fundamental to the construct, and no *a priori* evidence suggests differential importance (Streiner et al., 2014).

**Table 4:** Physical literacy assessment scale for primary and secondary physical educators.

Domains	Items
PA	I enjoy teaching students sports knowledge.
	I believe that through physical education, students can master sports skills.
	I pay close attention to the psychological and emotional changes that students undergo during the learning process.
PS	During lessons, I can demonstrate sporting techniques to my students.
	I perform physical activities with my students during lessons.
	I will take the initiative to step up my training in specific physical skills.
	I will work on improving my teaching skills during my spare time.
	I will take the initiative to organize sports competitions at school and within classes.
	In my spare time, I participate in sports competitions.
PC	I believe that physical education lessons should aim to cultivate students’ physical literacy.
	I know which methods and approaches can improve my physical literacy.
	I will explain the fundamental knowledge of physical education to my students (such as injury prevention and balanced diet).
	I understand the requirements set out in the National Curriculum Guidelines for Physical Education (such as the concept of physical literacy).
PCD	I am able to demonstrate my physical literacy through physical activity.
	I place great emphasis on fostering a lifelong exercise habit among my students.
	I possess the habit of exercise regularly.
	I will incorporate content related to ethics and spirit into my physical education lessons.
	In my physical education lessons, I never use language that might hurt my students’ feelings.
	I will treat students equally, whether they are top or bottom.
	I will ask students for their personal situation (study and life, etc.).

The PLAS-PET consists of 20 items scored from 1 to 5 (There are five options in the Degree of Agreement: 1 = Strongly disagree, 2 = Disagree, 3 = Neutral, 4 = Agree, 5 = Strongly agree). The total score range is 1–5, with higher scores indicating higher perceived PL. PA, Physical affectivity; PS, Physical skill; PC, Physical cognition; PCD, Physical conduct.

### 3.6 Results of confirmatory factor analysis

Based on the results of the EFA, this study constructed a first-order, four-factor structural equation model. Subsequently, data from Subsample 2 (*n* = 390) were imported, and the structural equation model was tested and compared using the maximum likelihood estimation method in AMOS 24.0. Figure 2 presents the first-order four-factor structural equation model of physical education teachers’ PL. This model consists of four first-order factors (physical affectivity, physical skill, physical cognition, and physical conduct). In Figure 2, the rectangles (Q1…Q20) represent the 20 measurement items, and the ellipses represent the latent variables. The values on each unidirectional arrow are standardized factor loadings ranging from 0.75 to 0.87. All values exceeded the acceptance criterion of 0.50, indicating that the observed variables provided good explanatory power for their respective latent variables.

**Figure 2:**
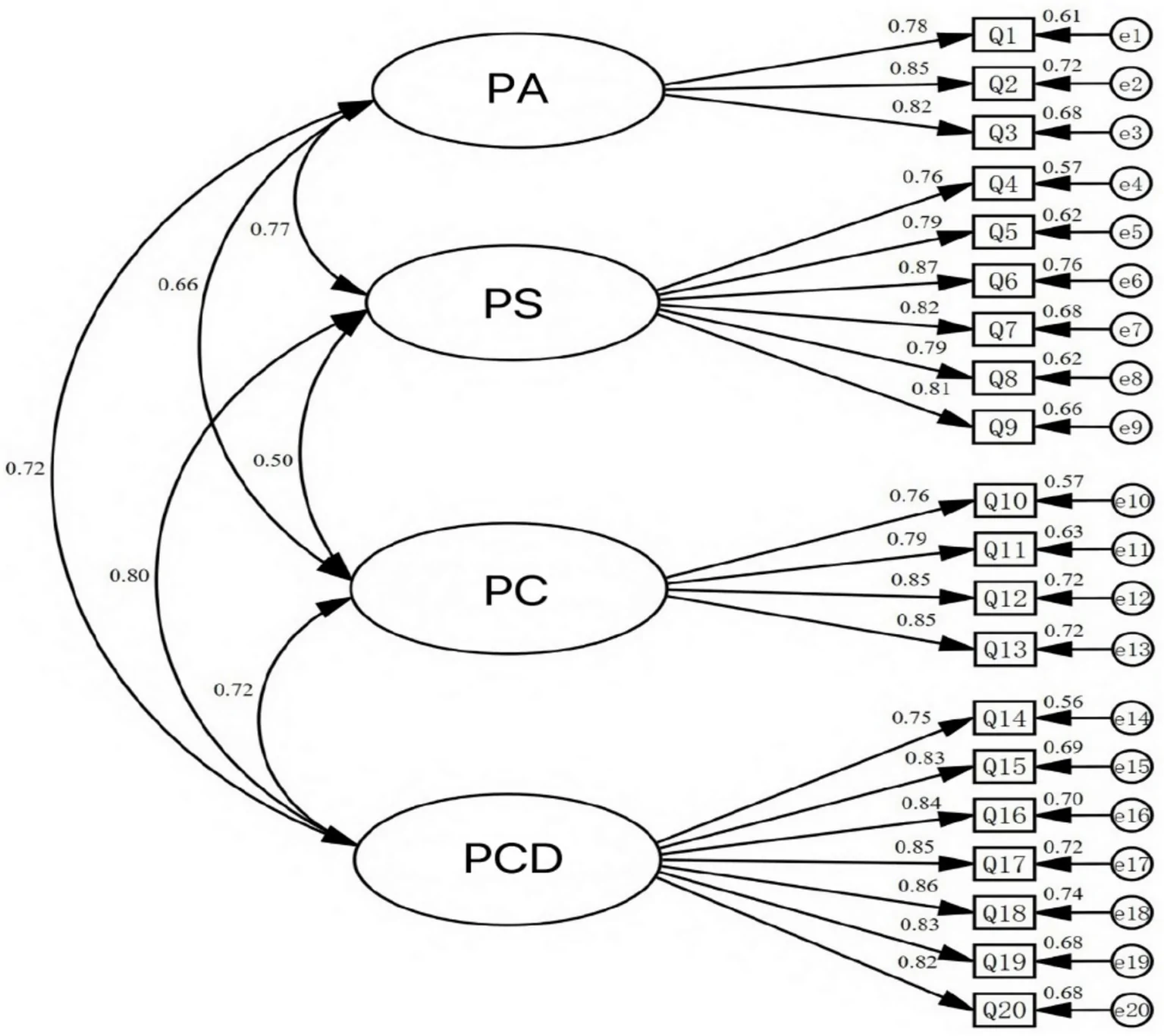
A first-order four-factor structural equation model of physical literacy for primary and secondary physical educators.

The fit results for the structural equation model of physical education teachers’ PL in primary and secondary schools were as follows: RMSEA = 0.071, *χ*^2^/df = 3.116, GFI = 0.874, CFI = 0.935, IFI = 0.935, TLI = 0.925, and SRMR = 0.072. Although the GFI is slightly below the standard threshold, it is still acceptable, and the results of the CFA indicate that the structural model fits well (Table 5). These results indicate that the scale’s item design is reasonable and that the proposed first-order, four-factor model demonstrates acceptable fit to the data. Furthermore, these results demonstrate that the PLAS-PET possesses good construct validity and confirm the validity of the framework for PL among primary and secondary school physical educators.

**Table 5:** Results of confirmatory factor analysis.

Statistical test	Criteria or thresholds for adaptation	Model adaptation value
RMSEA	<0.08 (<0.05 excellent)	0.071
RMSEA 90% CI	<0.08	[0.064, 0.078]
GFI	>0.90	0.874
CFI	>0.90	0.935
IFI	>0.90	0.935
TLI	>0.90	0.925
SRMR	<0.08	0.072
*χ*^2^/df	Between 0 and 3 (<5 is acceptable)	3.116

### 3.7 Reliability test

The Cronbach’s alpha coefficient for the PLAS-PET was 0.958. The Cronbach’s alpha coefficients for the four subscales, physical affectivity, physical skill, physical cognition, and physical conduct were 0.850, 0.916, 0.891, and 0.940, respectively. These results demonstrate that the items on the PLAS-PET exhibit good internal consistency. The Spearman–Brown split-half reliability coefficient for the PLAS-PET was 0.911. The Spearman–Brown split-half coefficients for the four subscales, physical affectivity, physical skill, physical cognition, and physical conduct were 0.770, 0.921, 0.860, and 0.910, respectively (Table 6). Therefore, these results indicate that the PLAS-PET possesses good reliability. McDonald’s *ω* values computed from the CFA standardized parameters were 0.75 for PA, 0.86 for PS, 0.85 for PC, and 0.91 for PCD (Table 6). Scale-level *ω* was not computed because the CFA model was first-order, four-factor, and did not include a single general factor or a bifactor structure. A total *ω* would require a unidimensional, second-order, or bifactor model (McDonald, 2013; Rodriguez et al., 2016). These values indicate acceptable to excellent model-based reliability across domains (McDonald, 2013; Zinbarg et al., 2006).

**Table 6:** Reliability statistics.

Domains	Cronbach’s alpha	Spearman–Brown	McDonald’s *ω*	*N* of items
Scale	0.958	0.911	–	20
PA	0.850	0.770	0.75	3
PS	0.916	0.921	0.86	6
PC	0.891	0.860	0.85	4
PCD	0.940	0.910	0.91	7

PA, Physical affectivity; PS, Physical skill; PC, Physical cognition; PCD, Physical conduct.

### 3.8 Validity test

The I-CVI of the PLAS-PET (30 items) ranged from 0.800 to 1.000, with an S-CVI/UA of 0.800 and an S-CVI/Ave of 0.960. These results indicate that the items in the scale effectively and accurately reflect the concept of PL among primary and secondary physical educators. Consequently, it can be concluded that the PLAS-PET has good content validity. Upon analysis, the standard factor loadings for all items on the PLAS-PET were greater than 0.500. The AVEs for the subscales, physical affectivity, physical skill, physical cognition, and physical conduct were 0.670, 0.651, 0.661, and 0.682, respectively, with CR values of 0.859, 0.918, 0.886, and 0.938, respectively (Table 7). Therefore, these results indicate that the PLAS-PET possesses good convergent validity. For discriminant validity, the square roots of AVE were 0.818 for PA, 0.807 for PS, 0.813 for PC, and 0.826 for PCD (Table 8). All inter-factor correlations were below the corresponding square roots of AVE, providing preliminary evidence of discriminant validity (Fornell and Larcker, 1981).

**Table 7:** Convergent validity statistics.

Domains	Factor loading	AVE	CR
PA	0.783–0.849	0.670	0.859
PS	0.755–0.872	0.651	0.918
PC	0.758–0.850	0.661	0.886
PCD	0.747–0.859	0.682	0.938

PA, Physical affectivity; PS, Physical skill; PC, Physical cognition; PCD, Physical conduct.

**Table 8:** Discriminant validity statistics.

Domain	PA	PS	PC	PCD	√AVE
PA	–	0.77	0.66	0.80	0.818
PS	0.77	–	0.50	0.72	0.807
PC	0.66	0.50	–	0.72	0.813
PCD	0.80	0.72	0.72	–	0.826

PA, Physical affectivity; PS, Physical skill; PC, Physical cognition; PCD, Physical conduct.

## 4 Discussion

### 4.1 The value of assessment tools

In developing the PLAS-PET, this study drew on Whitehead’s conceptual framework of PL. Through a literature review and an analysis of the practical characteristics of frontline physical education teachers in primary and secondary schools, the study designed the domains and items to ensure that they were comprehensive. Subsequently, the Delphi expert consultation method was employed to optimize and refine the initial scale item descriptions. After the preliminary construction of the scale was completed, data were collected using a questionnaire survey. Item screening was conducted based on item analysis, domain classification was performed using EFA, and the validity of domain classification was tested through CFA. Ultimately, a PLAS-PET was developed, comprising four domains, physical affectivity, skill competence, physical cognition, and physical conduct, and consisting of 20 items. Research indicates that marked differences in pupils’ physical and psychological characteristics across educational stages have, to some extent, led to variations in the PL of physical education teachers (Liu et al., 2026; Qian et al., 2026). Using a single PL measurement tool to assess teachers across all levels could significantly reduce the reliability and accuracy of the results (Barnett et al., 2023). The development of the PLAS-PET tool has refined the applicability and specificity of current assessment tools for physical education teachers’ PL, thereby enhancing the reliability and validity of the measurement results. This tool clearly defines the scope of application for the teacher population, which constitutes a fundamental difference from the existing PPLI tool for measuring physical education teachers’ PL. In contrast to the PPLI (Sum et al., 2016), which comprises nine items and three domins, the PLAS-PET adopts a domain-based structure that covers affective, skill, cognitive, and conduct dimensions. This broader coverage may offer a more comprehensive assessment framework, although direct comparative evidence between the two instruments has not yet been established. Many scholars have pointed out that the development of assessment tools must be based on a series of indicators (domains), and the scientific validity of these evaluation indicators (domains) directly determines the scientific validity of the assessment tool (Boateng et al., 2018; Haynes et al., 1995). From this perspective, the PLAS-PET meets the basic requirements for the development of measurement tools and can serve as an effective instrument for measuring the PL of teachers.

### 4.2 A conceptual discussion of assessment tools

This study makes a conceptual contribution by situating PL at the primary and secondary physical educator level. In this study, primary and secondary physical educators’ PL is defined as the motivation, confidence, physical competence, knowledge and understanding, and responsible physical activity behaviors that enable physical educators to value, participate in, and model lifelong physical activity and to enact these attributes in school physical education. This definition distinguishes primary and secondary physical educators’ PL from related but distinct constructs such as teaching efficacy, pedagogical content knowledge, and students’ PL (Mota et al., 2023). The division of domains in this scale aligns with the discussions by Whitehead and other scholars regarding the multidimensional nature of PL, while also addressing the shortcoming of existing measurement tools for physical education teachers’ PL (such as the PPLI), which do not sufficiently emphasize the conceptual essence of PL. Research indicates that current physical education teachers still lack a clear understanding of the conceptual meaning of PL (Durden-Myers et al., 2018; Robinson et al., 2018), resulting in teaching objectives, content, and methods in physical education classes that deviate from the goals of fostering PL. The four domains included in the PLAS-PET reflect the essence of PL to some extent. This can help physical education teachers enhance their awareness and understanding of PL and guide their teaching practices more effectively. From the perspective of measurement tools for PL, existing research has primarily focused on the development of measurement tools for children’s PL (Blanchard et al., 2020), the construction of models for adolescents’ PL (Pajkić et al., 2026), and self-assessment tools for adults’ PL (Matsunaga et al., 2025). However, measurement tools specifically designed for primary and secondary physical educators have been largely neglected. It is important to note that PL is, in essence, a lifelong journey and by no means a single experience or perception at a specific moment in time (Simpson et al., 2024). This may also indicate that there are differences in PL among various groups; therefore, when using assessment tools, attention must be paid to their relevance and suitability. In addition, the development of PL in early stage primary and secondary school students (children) is widely regarded as the foundation for lifelong physical activity, holding significant importance for ensuring physical and mental health throughout the entire life cycle and maintaining social stability (Cale and Harris, 2018). Research has shown that teachers’ PL can influence students’ health through role modeling (Liu et al., 2025; Yan et al., 2024). The level of PL among physical education teachers may indirectly affect students’ participation in physical activity and health outcomes through teaching behaviors, role modeling, and the classroom environment (Yan et al., 2024). The “physical conduct” (PCD) domain of the PLAS-PET includes items such as “fostering lifelong exercise habits in students” and “integrating moral and spiritual content into PE lessons.” These teacher behaviors may be key mechanisms linking teachers’ PL to students’ health outcomes. Consequently, whether primary and secondary physical educators possess the concept of PL and maintain a high level of PL is of critical importance, as these are essential prerequisites for fostering PL among primary and secondary school students. In summary, the development of a scientific and practical PL assessment tool for primary and secondary physical educators undoubtedly holds significant theoretical and practical value.

### 4.3 Methodological considerations and psychometric evidence

The PLAS-PET demonstrated good reliability and validity. To ensure the scale’s reliability and validity, it underwent quantitative testing in the following five stages: (1) Expert evaluation to ensure content validity. The I-CVI of the PLAS-PET ranged from 0.800 to 1.000, with an S-CVI/UA of 0.800 and an S-CVI/Ave of 0.960. These results indicate that the experts had a high level of acceptance of the scale’s evaluation indicators and that their opinions were relatively consistent (Polit et al., 2007). (2) Item analysis was conducted to ensure item representativeness. The critical ratios for the items ranged from 8.523 to 29.443, with all items reaching a highly significant level (*p* < 0.001). These results indicate that the measurement items can effectively differentiate between high-scoring and low-scoring respondents, the measurement items have good representativeness. (3) Exploratory and confirmatory factor analyses were conducted to ensure construct validity. EFA showed that the factor loadings of each item on its corresponding common factor were all >0.4, and no cross-factor multiple loadings were observed, indicating that the PLAS-PET has a clear factor structure. CFA was used to test the rationality of the domain divisions. The results indicated that the model fit indices of the structural equation model for the scale were acceptable, suggesting that the scale has good construct validity. (4) To ensure the scale’s internal consistency reliability. Both Cronbach’s *α* and the Spearman–Brown split-half reliability coefficients for the PLAS-PET were >0.7, indicating that the scale has good internal consistency (Cronbach, 1951; Charter, 2000), and thus, possesses good reliability. (5) A convergent validity analysis was conducted to ensure the clustering of similar items. The CR values for the PLAS-PET were all >0.7, and the AVE values were all >0.5, indicating that the developed scale possessed good convergent validity (Hair et al., 2019). The Fornell–Larcker criterion confirms that the discriminant validity of the PLAS-PET is acceptable. In summary, the PLAS-PET demonstrates more convincing reliability and validity, as it does not rely on a single statistical method to test these properties. A high-quality measurement tool typically needs to simultaneously meet the requirements of other indicators (Han et al., 2023), such as reliability (e.g., Spearman–Brown split-half reliability coefficient and Cronbach’s *α* coefficient) and validity (e.g., construct validity and convergent validity). In light of this, the PLAS-PET provides both the practical tools and the theoretical basis needed to assess the current state of PL among these teachers effectively and to formulate targeted strategies to promote their PL development.

## 5 Limitations and future directions

This study utilized a sampling approach to conduct a preliminary validation of a self-report assessment tool for physical educators’ PL; several limitations should be acknowledged. First, the study was conducted within the context of Chinese primary and secondary physical education. Cultural norms, curriculum guidelines, and teacher–student role expectations may shape how teachers interpret items related to ethical conduct, lifelong exercise, and role modeling. Therefore, the generalizability of the PLAS-PET to other cultural and educational settings remains untested. Second, several psychometric properties were not examined. Test–retest reliability, measurement invariance across gender, school level, and region, cross-cultural validation, and comparisons with independent assessments such as accelerometry, fitness tests, or observed teaching behavior were not conducted. Future research should address test–retest reliability, measurement invariance, cross-cultural validation, and independent assessments before the PLAS-PET is used for large-scale or high-stakes purposes.

## 6 Conclusion

The PLAS-PET developed in this study possesses a certain degree of scientific rigor and reliability and can serve as an effective tool for measuring the PL of primary and secondary school physical education teachers.

## Data Availability

The original contributions presented in the study are included in the article/Supplementary material, further inquiries can be directed to the corresponding authors.
