# Supplementary material for: What about the teachers? Developing and validating a physical literacy assessment scale for primary and secondary physical educators
Source: Front Psychol. 2026 Oct 29;17:1989587. doi: 10.3389/fpsyg.2026.1989587 (PMC13754680; doi:10.3389/fpsyg.2026.1989587)
Supplement: Supplementary file 1 [file Supplementary_file_1.docx]

**Appendix**

**Appendix 1**

*Table S1. Initial 32-item pool, sources, wording/adaptation notes, and rationale based on the physical literacy definition*

| Domain | Item ID | Original item | Source/adaptation | adaptation note | Rationale based on the PL definition |
| --- | --- | --- | --- | --- | --- |
| PA | PA01 | I enjoy teaching students sports knowledge. | Adapted from PPLI and teacher affect literature. | Retained. | Captures emotional engagement in teaching physical activity. Directly measures teachers’ own motivation in physical activity, a core affective PL attribute. |
|  | PA02 | I believe that through physical education, students can master sports skills. | Adapted from PL affective/confidence literature. | Retained. | Represents confidence in the value of PE and emotional commitment to student skill development. |
|  | PA03 | I pay close attention to the psychological and emotional changes that students undergo during the learning process. | Adapted from PPLI and teacher affect literature. | Retained. | Reflects emotional engagement and sensitivity in teaching, an affective enactment of PL. |
|  | PA04 | I feel confident when participating in physical activities. | Adapted from Whitehead’s affective domain. | Removed. | Directly measures teachers’ own confidence in physical activity, a core affective PL attribute. |
|  | PA05 | I enjoy participating in physical activity in my physical education. | Adapted from Whitehead’s affective domain. | Removed. | Directly measures enjoyment of physical activity, a core affective PL attribute. |
|  | PA06 | In physical education, I am motivated to maintain a physically active lifestyle. | Adapted from Whitehead’s affective domain. | Removed. | Directly measures motivation for lifelong physical activity, a core affective PL attribute. |
|  | PA07 | I frequently inquire about students’ academic and personal lives. | Generated by expert panel. | Removed after Delphi review. | Measuring teachers’ emotional involvement in teaching contexts is a manifestation of PL in the emotional dimension. |
|  | PA08 | I feel emotionally engaged when I am physically active. | Adapted from Whitehead’s affective domain. | Removed. | Measures emotional engagement in physical activity, a core affective PL attribute. |
| PS | PS01 | During lessons, I can demonstrate sporting techniques to my students. | Adapted from PL skill domain and PPLI. | Retained. | Captures movement competence and demonstration, which are central to the skill domain of PL. |
|  | PS02 | I perform physical activities with my students during lessons. | Adapted from PL skill domain. | Retained. | Captures active participation and role modeling in physical activity. |
|  | PS03 | I will take the initiative to step up my training in specific physical skills. | Adapted from PL skill domain. | Retained. | Captures self-directed improvement in physical skill, a core skill-domain attribute. |
|  | PS04 | I will work on improving my teaching skills during my spare time. | Adapted from teacher competency literature. | Retained. | Captures movement competence and demonstration, which are central to the skill domain of PL. |
|  | PS05 | I will take the initiative to organize sports competitions at school and within classes. | Adapted from PL skill/behavior literature. | Retained. | Captures active participation and organizational physical activity behavior. |
|  | PS06 | In my spare time, I participate in sports competitions. | Adapted from PL skill/behavior literature. | Retained. | Captures ongoing participation in sport and physical activity, which are central to the skill domain of PL. |
|  | PS07 | I demonstrate technical movements in every class. | Generated by expert panel. | Removed after Delphi review as redundant with PS01 and because it violated item independence. | Captures movement competence and demonstration, which are central to the skill domain of PL. |
|  | PS08 | I demonstrate technical movements to students when correcting incorrect techniques. | Generated by expert panel. | Removed. | Captures movement competence and demonstration, which are central to the skill domain of PL. |
| PC | PC01 | I believe that physical education lessons should aim to cultivate students’ physical literacy. | Adapted from PL cognitive domain. | Retained. | Captures knowledge and understanding of PL as a curriculum goal. |
|  | PC02 | I know which methods and approaches can improve my physical literacy. | Adapted from pedagogy literature. | Retained. | Capturing teachers’ understanding of PL is a reflection of their understanding of PL. |
|  | PC03 | I will explain the fundamental knowledge of physical education to my students (such as injury prevention and balanced diet). | Adapted from PL cognitive domain. | Retained. | Captures knowledge of health, safety, and physical activity, a core cognitive PL attribute. |
|  | PC04 | I understand the requirements set out in the National Curriculum Guidelines for Physical Education. | Adapted from curriculum literature. | Retained. | Captures curriculum knowledge, part of teacher-contextualized cognitive PL. |
|  | PC05 | In order to cultivate students’ physical literacy, I can use different teaching methods. | Suggested by Delphi expert. | Removed. | Captures pedagogical knowledge, which is part of teacher-contextualized cognitive PL. |
|  | PC06 | I know the rules and tactics of the sports I teach. | Adapted from PL cognitive domain. | Removed. | Captures sport-specific knowledge, a component of physical competence and knowledge. |
|  | PC07 | I understand how physical activity affects health and well-being. | Adapted from PL cognitive domain. | Removed. | Captures health-related knowledge, a core cognitive PL attribute. |
|  | PC08 | I know how to assess students’ physical literacy. | Adapted from PL assessment literature. | Removed. | Captures knowledge and understanding of PL as a curriculum goal. |
| PCD | PCD01 | I am able to demonstrate my physical literacy through physical activity. | Adapted from PL behavioral/ethical domain. | Retained. | Directly measures teachers’ own regular physical activity behavior, a core behavioral PL attribute. |
|  | PCD02 | I place great emphasis on fostering a lifelong exercise habit among my students. | Adapted from PL behavioral domain. | Retained. | Captures promotion of lifelong physical activity, a behavioral enactment of PL. |
|  | PCD03 | I possess the habit of exercise regularly. | Adapted from PL behavioral domain. | Retained. | Directly measures teachers’ own regular physical activity behavior, a core behavioral PL attribute. |
|  | PCD04 | I will incorporate content related to ethics and spirit into my physical education lessons. | Adapted from PL behavioral/ethical domain. | Retained. | Captures responsible and ethical behavior. |
|  | PCD05 | In my physical education lessons, I never use language that might hurt my students’ feelings. | Adapted from teacher ethics literature. | Retained. | Captures responsible and ethical behavior. |
|  | PCD06 | I will treat students equally, whether they are top or bottom. | Adapted from teacher ethics literature. | Retained. | Captures equity and fairness, captures responsible and ethical behavior. |
|  | PCD07 | I will ask students for their personal situation (study and life, etc.). | Adapted from teacher care literature. | Retained. | Captures care for students, captures responsible and ethical behavior. |
|  | PCD08 | I take responsibility for students’ safety during physical activities. | Suggested by Delphi expert. | Removed. | Captures safety responsibility, captures responsible and ethical behavior. |

*****PL = Physical literacy. PA = Physical affectivity, PS = Physical skill, PC = Physical cognition, PCD = Physical conduct.

**Appendix 2**

*Table S2. Mean, SD, and CV of initial items*

| Item | Domain | Mean | SD | CV |
| --- | --- | --- | --- | --- |
| Q1 | PA | 4.71 | 0.47 | 0.10 |
| Q2 | PA | 4.64 | 0.50 | 0.11 |
| Q3 | PA | 4.79 | 0.43 | 0.09 |
| Q4 | PS | 4.71 | 0.47 | 0.10 |
| Q5 | PS | 4.64 | 0.50 | 0.11 |
| Q6 | PS | 4.57 | 0.51 | 0.11 |
| Q7 | PS | 4.50 | 0.65 | 0.14 |
| Q8 | PS | 4.43 | 0.65 | 0.15 |
| Q9 | PS | 4.29 | 0.73 | 0.17 |
| Q10 | PC | 4.50 | 0.65 | 0.14 |
| Q11 | PC | 4.71 | 0.47 | 0.10 |
| Q12 | PC | 4.64 | 0.50 | 0.11 |
| Q13 | PC | 4.57 | 0.51 | 0.11 |
| Q14 | PCD | 4.79 | 0.43 | 0.09 |
| Q15 | PCD | 4.71 | 0.47 | 0.10 |
| Q16 | PCD | 4.64 | 0.50 | 0.11 |
| Q17 | PCD | 4.57 | 0.51 | 0.11 |
| Q18 | PCD | 4.50 | 0.65 | 0.14 |
| Q19 | PCD | 4.29 | 0.73 | 0.17 |
| Q20 | PCD | 4.36 | 0.74 | 0.17 |
| Q21 (PA04) | PA | 4.50 | 0.65 | 0.14 |
| Q22 (PA05) | PA | 4.43 | 0.65 | 0.15 |
| Q23 (PA06) | PA | 4.36 | 0.74 | 0.17 |
| Q24 (PA08) | PA | 4.29 | 0.73 | 0.17 |
| Q25 (PS08) | PS | 4.36 | 0.74 | 0.17 |
| Q26 (PC05) | PC | 4.79 | 0.43 | 0.09 |
| Q27 (PC06) | PC | 4.50 | 0.65 | 0.14 |
| Q28 (PC07) | PC | 4.29 | 0.73 | 0.17 |
| Q29 (PC08) | PC | 4.43 | 0.65 | 0.15 |
| Q30 (PCD08) | PCD | 4.43 | 0.65 | 0.15 |

*PA = Physical affectivity, PS = Physical skill, PC = Physical cognition, PCD = Physical conduct.

**Appendix 3**

*Table S3. Results of Exploratory Factor Analysis (Deleted items)*

| Domains | Items | F1 | F2 | F3 | F4 |
| --- | --- | --- | --- | --- | --- |
| PA | Q21 (PA04) | .412 | .205 | .118 | .093 |
| PA | Q22 (PA05) | .388 | .301 | .144 | .102 |
| PA | Q23 (PA06) | .465 | .176 | .203 | .119 |
| PA | Q24 (PA08) | .433 | .122 | .267 | .188 |
| PS | Q25 (PS08) | .201 | .472 | .113 | .156 |
| PC | Q26 (PC05) | .134 | .198 | .441 | .212 |
| PC | Q27 (PC06) | .098 | .254 | .398 | .301 |
| PC | Q28 (PC07) | .176 | .143 | .476 | .189 |
| PC | Q29 (PC08) | .223 | .112 | .457 | .204 |
| PCD | Q30 (PCD08) | .187 | .165 | .209 | .483 |

*****PA = Physical affectivity, PS = Physical skill, PC = Physical cognition, PCD = Physical conduct.

**Appendix 4**

*Table S4. Item-total Correlations for the 30 Initial Items*

| Item | Domain | Corrected item-total correlation |
| --- | --- | --- |
| Q1 | PA | .68 |
| Q2 | PA | .72 |
| Q3 | PA | .75 |
| Q4 | PS | .65 |
| Q5 | PS | .70 |
| Q6 | PS | .69 |
| Q7 | PS | .62 |
| Q8 | PS | .60 |
| Q9 | PS | .74 |
| Q10 | PC | .67 |
| Q11 | PC | .66 |
| Q12 | PC | .77 |
| Q13 | PC | .78 |
| Q14 | PCD | .58 |
| Q15 | PCD | .71 |
| Q16 | PCD | .69 |
| Q17 | PCD | .70 |
| Q18 | PCD | .64 |
| Q19 | PCD | .63 |
| Q20 | PCD | .61 |
| Q21 (PA04) | PA | .52 |
| Q22 (PA05) | PA | .48 |
| Q23 (PA06) | PA | .55 |
| Q24 (PA08) | PA | .46 |
| Q25 (PS08) | PS | .51 |
| Q26 (PC05) | PC | .49 |
| Q27 (PC06) | PC | .44 |
| Q28 (PC07) | PC | .53 |
| Q29 (PC08) | PC | .47 |
| Q30 (PCD08) | PCD | .50 |

*****PA = Physical affectivity, PS = Physical skill, PC = Physical cognition, PCD = Physical conduct.
